# Etude des facteurs de risque du retard de croissance intra-utérin à Lubumbashi

**DOI:** 10.11604/pamj.2013.14.4.1798

**Published:** 2013-01-03

**Authors:** Jules Ngwe Thaba Moyambe, Pierre Bernard, Faustin Khang'Mate, Albert Mwembo Tambwe A Nkoy, Faustin Chenge Mukalenge, Daudet Makanda, Eugene Twite, Arthur Munkana Ndudula, Cham Lubamba, Arnauld Kabulu Kadingi, Mutach Kayomb, Prosper Kalenga Muenze Kayamba

**Affiliations:** 1 Département de Gynécologie et Obstétrique, Faculté de médecine de l'Université de Lubumbashi, RD Congo; 2 Faculté de médecine, Université Catholique de Louvain, Bruxelles, Belgique; 3 Faculté de médecine Vétérinaire, Université de Lubumbashi, RD Congo

**Keywords:** Retard de croissance intra-utérin, facteurs de risque, Lubumbashi, RDC, Intrauterine growth restriction, risk factor, Lubumbashi, Democratic Republic of Congo

## Abstract

**Introduction:**

Dans notre milieu, il n'existe aucune politique de prévention du Retard de Croissance Intra-Utérin (RCIU) clairement défini. L'objectif de ce travail était d'identifier les facteurs de risque de RCIU afin de proposer une stratégie de lutte contre cette pathologie en agissant surtout sur des facteurs pouvant faire l'objet d'une action préventive.

**Méthodes:**

Une étude cas-témoins a été menée dans 11 centres hospitaliers de Lubumbashi en République Démocratique du Congo, de Janvier 2010 à Juin 2011, dans le but d'identifier les facteurs de risque du retard de croissance intra-utérin (RCIU). Au total 420 gestantes (cas et témoins) avec grossesse monofoetale d'au moins 24 semaines d'aménorrhée ont été inclues dans l'étude. Les cas correspondaient aux gestantes dont le poids du fœtus était resté inférieur au 10 eme percentile des courbes de référence d'Alexander, après 2 échographies successives réalisées à intervalle de 4 semaines. Les témoins correspondaient aux gestantes dont le poids du fœtus était supérieur ou égal au 10 eme percentile de mêmes courbes. A chaque cas a été apparié un témoin de même parité porteur d'une grossesse de même âge.

**Résultats:**

L'analyse univariée a identifié comme facteurs de risque: la taille maternelle.

**Conclusion:**

L'amélioration du niveau socio-économique des populations, la lutte contre le paludisme et les consultations prénatales mieux organisées couplées à une meilleure éducation sanitaire et nutritionnelle peuvent contribuer sensiblement à la réduction de la fréquence du RCIU à Lubumbashi.

## Introduction

Le retard de croissance intra-utérin (RCIU) désigne une réduction pathologique du profil de croissance attendu d'un fœtus habituellement en relation avec un problème survenu in-utero [1]. De cette réduction de croissance peut résulter une hypotrophie qui est définie par un poids inférieur au 10ème percentile dans une population donnée de même âge gestationnel [2]. La notion d'hypotrophie se réfère au faible poids de naissance; pour une grossesse à terme, l'hypotrophie correspond à un poids de naissance inférieur à 2500 grammes selon l'OMS [3]. Le RCIU et l'hypotrophie sont souvent liés constituant ainsi un problème majeur de santé de par leur forte association avec la morbidité et la mortalité périnatales.

En effet, le risque de décès néonatal et infantile pour un hypotrophe en RCIU est 2 à 3 fois plus important que pour un nouveau-né eutrophe. Outre le risque pour sa survie immédiate, le RCIU prédispose le nouveau-né hypotrophe à des déficits cognitifs, à des moindres capacités physiques, à un risque des maladies tout au long de la vie et même à des maladies chroniques liées à l'alimentation [4]. A terme le nouveau-né en RCIU avec faible poids de naissance présente des scores d'Apgar et des pH néonataux inférieurs à ceux d'autres nouveau-nés [5]. A prématurité identique, les nouveau-nés en RCIU présentent plus des complications néonatales sévères que les nouveau-nés sans RCIU avec 2 fois plus d'entérocolite ulcéro-nécrosante [6]. En 2004, l'USSCN (United Nations system Standing Committee on Nutrition) [7] avait estimé à environ 30 millions le nombre d'enfants qui naissent chaque année avec une insuffisance pondérale et d'après l'OMS, 3, 4 millions en sont morts en 2002 [8]. Les pays les plus touchés sont les pays d'Afrique sub-saharienne et les pays d'Asie du Sud-Est (particulièrement l'Inde) où les cas d'insuffisance pondérale dépassent respectivement 15 et 30% de naissances. En Afrique, l'incidence du RCIU se situe entre 10 et 20% des naissances [9, 10], alors que dans les pays industrialisés, elle oscille entre 2 et 5% [11]. En R.D.Congo, une étude menée dans la Province du Maniema par Milabyo [12] à Kipaka dans la zone de santé rurale de Kunda et à Kama dans la zone de santé rurale de Kampene sur le faible poids de naissance a donné une prévalence de 27% à Kipaka et de 16, 4% à Kama.

Du fait de ses conséquences et de son taux élevé de morbi- mortalité périnatale, le RCIU constitue un véritable problème de santé publique. Dans notre milieu, il n'existe aucune politique de prévention du RCIU clairement défini. C'est pourquoi nous avons entrepris cette étude dans le but d'identifier les facteurs de risque de RCIU afin de proposer une stratégie de lutte contre cette pathologie en agissant surtout sur des facteurs pouvant faire l'objet d'une action préventive.

## Méthodes

Une étude cas-témoins a été réalisée dans 11 centres hospitaliers de la ville Lubumbashi, Chef-lieu de la province du Katanga en République Démocratique du Congo (RDC) de Janvier 2010 à Juin 2011. Les gestantes ayant des cycles menstruels réguliers, porteuses d'une grossesse monofoetale évolutive d'au moins 24 SA avec date de dernières règles connue, sans métrorragie au cours de la grossesse ont été inclues dans l'étude. Le cas a été défini comme toute gestante répondant aux critères d'inclusion et dont le poids du fœtus était resté inférieur au 10ème percentile de courbes de référence pour l'âge gestationnel d'Alexander et al. [13] à une deuxième échographie faite à quatre semaines d'intervalle de la première [14, 15]. Les courbes de référence pour l'âge gestationnel d'Alexander et al. [13] sont des courbes spécifiques à la race noire. Le témoin a été défini comme toute gestante répondant aux mêmes critères que le cas et dont le poids du fœtus était supérieur ou égal au 10e percentile de mêmes courbes. Les témoins ont été recrutés de manière séquentielle au ratio de 1 témoin pour 1 cas. A chaque cas a été apparié comme témoin la première gestante répondant aux critères définis, de la même institution hospitalière, de la même parité et porteuse d'une grossesse de même âge.

La taille minimale de l'échantillon a été calculée à l'aide du logiciel Epi Info 6 dans sa fonction Stat Cal pour une étude cas-témoin. Etant donné qu'il y a plusieurs facteurs de risque à étudier, nous avons choisi l'état nutritionnel de la gestante comme facteur pour calculer la taille minimale de l'échantillon. Pour évaluer l'état nutritionnel de la gestante, l'indicateur qui a été retenu est le périmètre brachial; nous avons considéré un PB inférieur à 24 cm comme étant associé à un risque de RCIU. N'ayant pas des données disponibles localement sur le RCIU et l'état nutritionnel, nous avons considéré l'étude de Nikiema et al. [16] qui estime à 50% dans les pays en développement la proportion des gestantes ayant un PB inférieur à 24 cm chez les témoins. L'Odds Ratio minimum attendu, appréciant le risque de RCIU associé à un état nutritionnel insuffisant est de l'ordre de 1, 8 d'après la littérature [2]. Nous avons donc calculé en consentant une erreur alpha de 0, 05 et une puissance de 80%, avec un ratio de un témoin pour un cas, le nombre minimum des sujets nécessaires pour l'étude à 201 cas et 201 témoins. Au total 210 paires des couples gestante-fœtus (cas et témoins) ont été retenues pour l'étude.

Les paramètres suivants ont été retenus pour l'étude: la parité; la taille maternelle; le sexe du fœtus; l'âge gestationnel; la tension artérielle maternelle (l'hypertension artérielle chez la gestante est définie par une TA ≥ 140/90mmhg); l'état nutritionnel (l'indicateur utilisé pour apprécier l'état nutritionnel est le périmètre brachial [17–19], un mauvais état nutritionnel de la femme enceinte est caractérisé par un périmètre brachial inferieur à 24 cm); le taux d'hémoglobine (l'anémie a été définie selon les critères de l'OMS par un taux d'hémoglobine (Hb) inférieur à 11g%, elle est sévère si le taux d'Hb est < 8 g%); l'âge maternel (est à risque s'il est inférieur à 18 ans ou supérieur à 35 ans [20]. L'espace intergénésique (dans cette étude, il est défini comme l'écart entre la date du dernier accouchement et le début de la grossesse actuelle c'est-à-dire la date du début de dernières règles; il est catégorisé en 3 classes: inférieur à12 mois, 12 à 24 mois et supérieur à 24 mois); le niveau d'études (selon Elshibly et al [21], le niveau d'études est défini comme bas s'il est inférieur ou égal à 8 ans d'études c'est-à-dire 2ème année du cycle secondaire et acceptable s'il est supérieur à 8 ans d'études); le poids fætal (a été estimé à l'échographie par la formule de Hadlock qui intègre les mensurations de la tête (BIP, PC), du tronc (PA) et de la longueur fémorale [22]; le risque professionnel (regroupe les professions ou les emplois nécessitant un effort physique: agricultrice, commerçante ambulante, etc.); l'exposition toxique pendant la grossesse (consommation d'alcool et exposition au monoxyde de carbone par utilisation du bois ou du charbon de bois pour la cuisine); le paludisme (paludisme symptomatique avec goutte épaisse positive); le statut marital (le statut de célibataire regroupe les veuves, les divorcées, et les femmes vivant seules; le statut de mariée regroupe les femmes mariées et les femmes vivant maritalement); le niveau socio- économique a été évalué selon le score de Traissac et al. [23] pour lequel le système de cotation repose sur trois paramètres: dépenses alimentaires journalières: moins de 10 000 F CFA ou 10 000 F CFA et plus; type d'habitat: en matériaux non durables ou durables; moyen de transport (véhicule): en commun ou personnel. On attribue une valeur de 1 ou de 2 à chacun de ces trois paramètres. La somme des valeurs aux trois paramètres constitue le score de Traissac et al [23], réparti en trois catégories: Score = 3, c'est le niveau socio-économique bas; score = 4 ou 5, c'est le niveau socio-économique moyen; score = 6, c'est le niveau socio-économique élevé. Ce score de Traissac a été réadapté en fonction du dollar américain (en 1997, 1 USD équivalait à 588 F CFA).

La saisie des données et l'analyse statistique ont été réalisées grâce au logiciel SPSS 18. Pour la recherche des facteurs de risque, les variables ont été regroupées par catégories. On a d'abord recherché par l'analyse uni variée l'association de chacune des variables avec le RCIU. Ensuite l'ajustement a été fait par la régression logistique. Seules les variables associées au RCIU ont été retenues dans le modèle final. Le seuil de signification était fixé à 0, 05 et les intervalles de confiance à 95%.

## Résultats

### Facteurs constitutionnels

La taille et l'âge de la gestante sont significativement associés au RCIU. En ce qui concerne l'âge de la gestante, par rapport à la classe de référence de 18-35 ans, le risque de RCIU est environ 3 fois plus élevé si l'âge de la gestante est inférieur à 18 ans (OR=2,88), mais non significatif pour les gestantes de plus de 35 ans (OR=1,21). Quant à la variable taille de la gestante, le risque de RCIU est presque 2 fois plus élevé lorsque la taille de la gestante est inférieure à 155 cm (OR=2, 43). Il n'y a pas d'association significative entre le sexe du fœtus et le RCIU (Tableau 1).

**Tableau 1:** Facteurs constitutionnels associés au retard de croissance intra-utérine à Lubumbashi

Facteurs de risque		Cas (RCIU) N=210		Témoins N=210		OR (IC95%)	p
		n	%	n	%		
Taille de la gestante (cm)	<155	29	13,81	13	6,2	2,43(1,17-5,10)	0,0092
	≥ 155	181	86,19	197	93,8		
Age de la gestante (ans)	< 18	19	9,05	7	3,3	2,88(1,12- 7,74) - 1,21(0,75-1,98)	0,026
	18 -35	141	67,14	160	76,2		
	> 35	50	23,81	43	20,5		
Sexe du fœtus	Masculin	111	52,9	102	48,6	1,19(0,79-1,77)	0,379
	Féminin	99	47,1	108	51,4		

RCIU: au retard de croissance intra-utérine; OR: Odd ratio

### Facteurs nutritionnels

Comme indicateur de l'état nutritionnel, le périmètre brachial est associé significativement au RCIU (p =0,00). Le risque de RCIU est 2 fois plus élevé en cas d'un état nutritionnel insuffisant c'est-à-dire lorsque le périmètre brachial est inférieur à 24 cm (OR=1,96).

Le taux d'hémoglobine est significativement associé au RCIU (p=0,02). Le risque de RCIU est 2 fois plus élevé lorsque le taux d'hémoglobine est inférieur à 8 g% c'est-à-dire en cas d'anémie sévère (OR=2, 28); par contre l'anémie modérée (taux d'hémoglobine 8-10,9%) n'est pas associée au RCIU (OR=0,91). (Tableau 2).

**Tableau 2:** Facteurs nutritionnels associés au retard de croissance intra-utérine à Lubumbashi

Facteurs de risque		Cas (RCIU) N=210		Témoins N=210		OR (IC95%)	P
		n	%	n	%		
Périmètre brachial (Cm)	<24	62	29,5	37	17,6	1,96(1,2-3,19)	0,00
	≥ 24	148	70,5	173	82,4		
Hémoglobine (g%)	<8	37	17.6	18	8.6	2.28 (1.21-4.34) 0.91 (0.61-1.36)	0.02
	8-10.9	104	49.5	109	51.9		
	≥ 11	69	32.9	83	39.5		

**RCIU:** au retard de croissance intra-utérine; OR: Odd ratio

### Exposition toxique

Concernant les variables toxiques, l'exposition au monoxyde de carbone (CO) par l'utilisation du bois et de charbon de bois pour la cuisine (p=0,06) et la consommation d'alcool (p=0,49), ne sont pas associées significativement au RCIU (Tableau 3).

**Tableau 3:** Pathologies observées de la grossesse associée au retard de croissance intra-utérine à Lubumbashi

Facteurs de risque		Cas (RCIU) N=210		Témoins N=210		OR (IC 95%)	p
		n	%	n	%		
Hypertension artérielle	Oui	41	19,5	19	9	2,44 (1,32-4,55)	0,02
	Non	169	80,5	191	91		
Paludisme	Oui	87	41,4	56	26,7	1,95 (1,26-3,00)	0,00
	Non	123	58,6	154	73,3		

### Facteurs socio-économiques

En ce qui concerne les variables socio-économiques, le niveau socio-économique (p=0,00) et l'espace inter génésique (p=0,02) sont significativement associés au RCIU. Le risque de RCIU est 2 fois plus élevé lorsque l'espace inter génésique est inférieur à 12 mois (OR=1,92), et il est environ 3 fois plus élevé en cas d'un bas niveau socio-économique (OR=2,73). Par contre ni le niveau d'études (p=0,12), ni le risque professionnel (p=0,36) et ni le statut marital (p=0,61) ne sont associés significativement au RCIU (Tableau 4).

**Tableau 4:** Facteurs socio- économiques associés au retard de croissance intra-utérine à Lubumbashi

Facteurs de risque		Cas (RCIU) N=210		Témoins N=210		OR (IC95%)	p
		n	%	n	%		
Niveau d'études	Bas	60	28,6	46	21,9	1,43(0,89-2,28)	0,12
	Acceptable	150	71,4	164	78,1		
Niveau socio-économique	Bas	98	46,7	51	24,3	2,73(1,76-4,23)	0,00
	Moyen	72	34,3	88	41,9	0,72(0,48-1,10)	
	Élevé	40	19	71	33,8		
Risque professionnel	Oui	55	26,2	47	22,4	1,23(0,77-1,97)	0,36
	Non	155	73,8	163	77,6		
Statut marital	Célibataire	21	10	18	8,6	0,84(0,41-1,71)	0,61
	Mariée	189	90	192	81,4		
Espace intergénésique (mois)		**Cas (RCIU) N=187**		**Témoins N=187**			
		**N**	**%**	**n**	**%**		0,01
	<12	35	18,7	20	10.7	1,92(1,02-3,63)	
	12-24	75	40,1	100	53	0,58(0,38-0,90)	
	>24	77	41,2	67	36.3		

### Pathologies observées pendant la grossesse

S'agissant des pathologies observées pendant la grossesse, il y a une association significative de l'hypertension artérielle avec le RCIU (p=0,02). Le risque pour une gestante hypertendue d'avoir un fœtus en RCIU est 2 fois plus élevé que pour une gestante non hypertendue (OR=2,44). Le paludisme est significativement associé au RCIU (p=0, 00). Le risque de RCIU est 2 fois plus élevé chez une gestante paludéenne que chez une gestante non impaludée (OR=1,95). (Tableau 5).

**Tableau 5:** Facteurs de risque associés au retard de croissance intra-utérine à Lubumbashi

Facteurs de risque		ORa (IC 95%)*	P
Taille de la gestante (cm)	< 155	3.939(1,614 - 9,613)	0.00
	≥155	1	
Niveau socio-économique	Bas	5.335 (2.578-11.042)	0.00
	Moyen	1.750 (0.935-3.272)	0.08
	Elevé	1	
Espace intergénésique (mois)	< 12	1.234(0.596-2.556)	0.57
	12-24	0.495(0.291-0.840)	0.01
	> 24	1
HTA	Oui	4.647 (2.250-9.596)	0.00
	Non	1	
Paludisme	Oui	2.268 (2.250-4.202)	0.01
	Non	1	
Périmètre brachial (cm)	< 24	0.968 (0.461-2.034)	0.93
	≥24	1
Hémoglobine (g%)	< 8	0.678 (0.212 - 2.161)	0.51
	8-10,9	0.900 (0.478 – 1.694)	0.74
	≥11	1	

* ORa=*Odds ratio* ajusté par rapport à d'autres variables au moyen de la régression logistique

### Analyse multivariée

Les variables pour lesquelles une association globale avec le RCIU a persisté au seuil de 0, 05 après recherche des effets conjoints des variables d'une même catégorie à l'analyse univariée sont: la taille de la gestante pour les facteurs constitutionnels, le niveau socio-économique et l'espace intergénésique pour les facteurs socio-économiques, le périmètre brachial et le taux d'hémoglobine pour les facteurs nutritionnels, le paludisme et l'HTA pour les pathologies observées pendant la grossesse.

Le modèle final de régression logistique comporte donc seulement 7 variables. L'effectif dans ce modèle est de 387 couples gestante-fœtus. En effet, les nullipares sont exclues de ce modèle car n'ayant pas d'espace intergénésique. Le Tableau 6 montre que le risque de RCIU est environ 4 fois plus élevé si la taille de la gestante est inférieure à 155 cm (OR=3,9), le risque de RCIU est 5 fois plus élevé si le niveau socio-économique est bas (OR=5,3). L'HTA est fortement associé au RCIU; le risque pour une gestante hypertendue d'avoir un fœtus RCIU est environ 5 fois plus élevé que pour une gestante non hypertendue (OR=4,6). Le paludisme symptomatique avec goutte épaisse positive reste significativement associé au RCIU; le risque de RCIU est 2 fois plus élevé chez la gestante paludéenne que chez une gestante impaludée (OR=2,3).

**Tableau 6:** Facteurs toxiques associés au retard de croissance intra-utérine à Lubumbashi

Facteurs de risque		Cas (RCIU) N=210		Témoins N=210		OR (IC95%)	P
		n	%	n	%		
CO*	Oui	186	88,6	197	93,8	0,51(0,24-1,08)	0,06
	Non	24	11,4	13	6,2		
**Alcool**	Oui	16	7,6	20	9,5	0,78(0,37-1.64)	0,49
	Non	194	92,4	190	90,5		

* monoxyde de Carbone

Par contre dans ce modèle, les effets du taux d'hémoglobine, de l'espace intergénésique et du périmètre brachial sur le RCIU deviennent non significatifs.

## Discussion

Les déterminants du RCIU sont multiples et intriqués. Dans cette étude, certains facteurs connus comme étant de risque de RCIU n'ont pas été recherchés; c'est le cas par exemple des anomalies chromosomiques. D'autres n'ont pas été signalés; c'est le cas par exemple de la consommation du tabac pendant la grossesse qui est un des principaux facteurs de risque de RCIU dans les pays industrialisés [24].

Pour ce qui est de la taille de la gestante, la petite taille de la gestante est généralement reconnue comme facteur d'hypotrophie fætale [25–27]. Par contre, pour Scott et Usher [28], ce facteur n'est pas significativement associé au RCIU. Dans notre étude nous avons observé une association significative entre la taille de la gestante et le RCIU. Le risque de RCIU est 2 fois plus élevé si la taille de la gestante est inférieure à 155 cm. En effet chez une gestante de petite taille il y a diminution ou faible volume d'éjection systolique cardiaque qui entraine une baisse de la perfusion utéroplacentaire avec transfert déficient des substances nutritives de la gestante vers le fœtus entrainant ainsi un RCIU [29].

En rapport avec l'âge maternel, le jeune âge maternel inférieur à 20 ans est communément associé au RCIU [26, 30]. Frisangho et al. [31] et Mafina-Mienandi et al. [32] ont rapporté le jeune âge maternel comme facteur de risque associé au RCIU. Par contre, certains auteurs n'ont pas trouvé de rapport entre l'âge maternel et la croissance fætale [25, 33]. Dans notre étude nous avons trouvé en analyse univariée qu'il y a une association significative entre l'âge maternel inférieur à 18 ans et le RCIU. Nous pensons que, les plus jeunes mères n'ont pas encore terminé leur croissance et qu'elles utilisent la grande partie des apports nutritionnels pour leur propre croissance au détriment de la croissance fætale.

L'état nutritionnel est considéré comme une condition résultant de l'équilibre entre l'ingestion des aliments et leur utilisation par l'organisme. Les indicateurs souvent utilisés pour mesurer cet état nutritionnel sont: l'indice de masse corporel avant la grossesse (IMC pregestationnel) et le périmètre brachial pendant ou en dehors de la grossesse. Dans cette étude très peu de gestantes ont pu déclarer, de façon fiable leurs poids, avant la grossesse si bien que nous avons utilisé le périmètre brachial pour évaluer leur état nutritionnel. Outre le fait qu'il était disponible chez toutes les gestantes, le périmètre brachial est un marqueur fidèle de l'état nutritionnel aussi bien avant que pendant la grossesse. Dans notre étude, le périmètre brachial était significativement lié au RCIU en analyse univariée et le risque de RCIU est 2 fois plus élevé si le périmètre brachial est inférieur à 24 cm. Notre observation rejoint celles de certaines études notamment les études de Ranakrishman [34], de Chabra et Bhandari [35] et de Meda et al. [18].

Concernant le paludisme et l'anémie: Dans les régions de transmission endémique, le paludisme pendant la grossesse est l'une des fréquentes causes de l'anémie maternelle et du faible poids de naissance. Il y a une interaction considérable entre le paludisme maternel et l'anémie grave au niveau de leurs effets sur le poids de naissance [36]. Le risque d'un faible poids de naissance est très élevé chez les gestantes avec une anémie grave [37]. L'anémie paludéenne aggrave généralement l'anémie carentielle habituellement observée chez les gestantes. L'anémie d'origine paludéenne serait due non seulement à l'hémolyse, mais aussi à d'autres troubles associés au paludisme notamment la carence martiale et/ou folique. La carence martiale peut se manifester à la suite d'une absorption insuffisante du fer lors des crises du paludisme (nausées, vomissements) et dans la forme grave qui s'accompagne de l'hémoglobinurie. Et en général aussi du fait qu'une quantité de fer est séquestrée dans le parasite sous forme d'hémozoïne [38]. Quant à la carence folique, celle-ci s'expliquerait par le fait que l'hémolyse stimule l'érythropoïèse et augmente les besoins en acide folique et finit par affecter le taux sérique de celui-ci [39]. Dans une étude faite en Inde, Singh et al [40] ont trouvé que 80% des gestantes paludéennes présentaient une anémie. Ngwe et al. [41] ont trouvé que 65, 5% des gestantes paludéennes présentaient une anémie. En zone d'endémie palustre stable, le paludisme au cours de la grossesse a pour conséquence l'anémie chez la mère et un faible poids de naissance chez le nouveau- né [42]. Landis [43] dans son étude, a constaté que le paludisme seul est faiblement associé au RCIU et qu'un effet indépendant du paludisme sur le RCIU est seulement observé chez les gestantes qui ont connu plus de trois infestations du paludisme au cours de la même grossesse. L'effet du paludisme maternel sur la croissance intra-utérine varie significativement avec l'état nutritionnel maternel. Ce risque est plus élevé chez les gestantes avec un mauvais état nutritionnel. L'anémie de la gestante et le RCIU présentent un intérêt particulier car ils sont en eux-mêmes les facteurs de vulnérabilité. Pour Fricker [44], les modifications physiologiques liées à la gestation rendent difficile l'interprétation du taux d'hémoglobine au-delà du premier trimestre de la grossesse. En effet, l'expansion physiologique du volume plasmatique est responsable d'une baisse du taux d'hémoglobine par hémodilution qui n'est pas à proprement parlé une anémie puisqu'elle n'est pas préjudiciable au bon déroulement de la grossesse tant que l'hémoglobine reste au-dessus de 9, 5 g%. Lorsque ce taux est inférieur à 8 g%, l'anémie augmente le risque d'hypotrophie fætale. Comme le signale Fricker [44], nous avons trouvé qu'un taux d'hémoglobine inférieur à 8 g% (anémie sévère) est associé significativement au RCIU. Meda et al. [18] dans une étude prospective incluant 247 gestantes, ont montré qu'un taux d'hémoglobine de la gestante supérieur à 9 g% ne présentait aucun risque ni pour la croissance fætale, ni pour la santé de la gestante. Quant à Wharton [26], il y a une nette relation entre le taux bas d'hémoglobine de la gestante et un faible poids de naissance et il s'interroge si dans ces conditions le taux bas d'hémoglobine est un simple marqueur des réserves maternelles réduites ou si cela a une influence directe sur la croissance fætale.

L'hypertension de la gestante est une cause connue de RCIU [25, 27, 33]. Dans notre étude l'hypertension artérielle de la gestante est significativement associée au RCIU. En effet l'HTA entraine une réduction de l'apport sanguin maternel au placenta, par une diminution du débit utéroplacentaire et cette diminution perturbe les échanges causant ainsi un RCIU.

En ce qui concerne le niveau socio-économique, nous avons trouvé dans notre étude, qu'il y a une association significative entre le bas niveau socio-économique de la gestante et le RCIU comme l'ont rapporté aussi les études de Hirve et Ganatra [45] et de Karim et al. [19]. En effet, le bas niveau socio-économique est souvent la cause d'un mauvais état nutritionnel qui entraine un RCIU [46].

Quant à l'espace intergénésique, l'intervalle bref entre les naissances a été évoqué par Rawlings et al. [47] et Mafina-Mienandi et al. [32] comme facteur de risque de RCIU, mais reste cependant controversé par Wessel et al. [48]. Dans notre étude un intervalle intergénésique inférieur à 12 mois est lié à un risque de survenu d'un RCIU. En effet les maternités nombreuses et trop rapprochées accroissent considérablement le risque d'anémie [49]. C'est pourquoi dans le souci de sauvegarder la santé de la mère et du fœtus, l'Unicef [49] recommande un espacement optimal de 36 mois.

## Conclusion

Le présent travail montre que le RCIU reste un sérieux problème de santé publique dans la ville de Lubumbashi et que parmi les déterminants les plus importants du RCIU on retrouve: la taille maternelle inférieure à 155 cm, le bas niveau socio-économique, le mauvais état nutritionnel, l'hypertension artérielle maternelle et le paludisme. Ces facteurs de risque devraient donc faire l'objet d'une lutte soutenue dans la prévention du RCIU. Dans l'ensemble, les consultations prénatales mieux organisées couplées à une meilleure éducation sanitaire et nutritionnelle peuvent contribuer sensiblement à la réduction de la fréquence du RCIU à Lubumbashi.
